# The fecal metabolomic signature of a plant-based (vegan) diet compared to an animal-based diet in healthy adult client-owned dogs

**DOI:** 10.1093/jas/skaf054

**Published:** 2025-02-27

**Authors:** Brooklynn D Liversidge, Sarah A S Dodd, Jennifer L Adolphe, Diego E Gomez, Shauna L Blois, Adronie Verbrugghe

**Affiliations:** Department of Clinical Studies, Ontario Veterinary College, University of Guelph, Guelph, ON, CanadaN1G 2W1; Department of Population Medicine, Ontario Veterinary College, University of Guelph, Guelph, ON, CanadaN1G 2W1; Department of Veterinary Biomedical Sciences, Western College of Veterinary Medicine, University of Saskatchewan, Saskatoon, CanadaS7N 5B4; Petcurean Pet Nutrition, Chilliwack, BC, CanadaV2R 5M3; Department of Clinical Studies, Ontario Veterinary College, University of Guelph, Guelph, ON, CanadaN1G 2W1; Department of Clinical Studies, Ontario Veterinary College, University of Guelph, Guelph, ON, CanadaN1G 2W1; Department of Clinical Studies, Ontario Veterinary College, University of Guelph, Guelph, ON, CanadaN1G 2W1

**Keywords:** alternative diets, canine, fecal metabolome, pet nutrition, plant-based ingredients

## Abstract

Despite the rising popularity of plant-based (vegan) diets for dogs, the metabolic effects of plant-based diets in dogs have not been thoroughly investigated. Evaluating the impact of a vegan diet on the fecal metabolome in dogs could offer valuable insight into the effects on gastrointestinal and overall health. This study evaluated the fecal metabolic signature of an experimental extruded vegan diet (PLANT) compared to a commercial extruded animal-based diet (MEAT) in healthy adult dogs. Sixty-one client-owned healthy adult dogs completed a randomized, double-blinded longitudinal study consisting of a 4-wk acclimation period, where all dogs received the MEAT diet, followed by a 12-wk experimental period where they either continued with the MEAT diet or switched to the PLANT diet. Fecal collections occurred at baseline (after 4-wk acclimation) and exit (after the experimental period). Fecal metabolites were quantified using ^1^H nuclear magnetic resonance spectroscopy. Multiple mixed model gamma linear regression was used to evaluate the association of metabolite concentration against age, sex, and body weight, along with an interaction between diet and time. Sixty-six metabolites were quantified. Only 2/66 metabolites differed between groups at baseline and within the MEAT diet group over time. In contrast, 46/66 metabolites differed in concentrations over time in response to feeding the PLANT diet. At the exit time-point, dogs fed the PLANT diet had increased metabolites related to carbohydrate fermentation, such as acetic (*P* < 0.01) and propanoic (*P* < 0.01) acid and increases in sugar metabolites when compared to the MEAT group. These findings indicate that the fecal metabolic signature of dogs fed a plant-based diet is distinct from dogs fed an animal-based diet, even if both diets have a similar nutrient profile and are processed similarly.

## Teaser

The study evaluated the impact of a plant-based (vegan) diet on the fecal metabolome of healthy adult dogs in comparison to a commercial animal-based diet. Sixty-one client-owned dogs completed a randomized, double-blinded longitudinal study where they either consumed an animal-based or plant-based diet exclusively for 12 wk. Fecal collections occurred at 2 time points (baseline and exit) and were analyzed using 1H NMR spectroscopy where results revealed changes in the fecal metabolic signature of dogs, highlighting distinct metabolic responses between plant and animal diets.

## Graphical Abstract

**Graphical Abstract:**
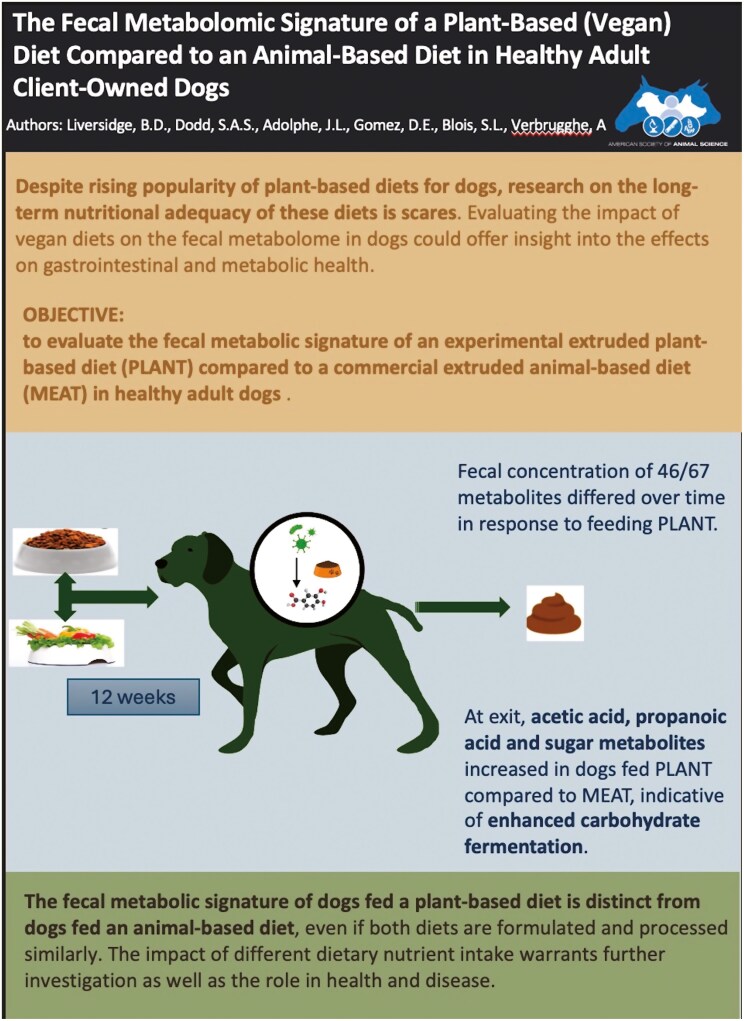

## Introduction

In most parts of the world, pet guardians view their dogs as family members, resulting in a strong human-animal bound, pets being seen as children and closely living with humans (Buff et al., 2014; Dodd et al., 2018; Carlos et al., 2020). It is no surprise that recently, human nutrition trends have shifted towards a more plant-based lifestyle (vegan or vegetarian), and a similar trend is seen in pet nutrition (Charlesbois et al., 2019). Vegan pet guardians may seek alternative plant-based dog food options as they prefer to avoid conventional animal-based dog food that does not align with their lifestyle (Laflamme et al., 2008; Buff et al., 2014; Dodd et al., 2018). Consequently, commercial plant-based diets marketed as nutritionally complete and balanced for dogs have gained popularity. However, the impact these diets have on dog health, specifically on the gastrointestinal tract remains a topic of debate among veterinarians and veterinary nutritionists (Wakefield et al., 2006; Rothgerber, 2013; Dodd et al., 2018). Research into the health effects of plant-based diets for dogs is emerging, with recent studies exploring hematological health outcomes, vitamin D and bone health, as well as gastrointestinal health including apparent total-tract nutrient digestibility and fecal microbiome (Dodd et al., 2023; Liversidge et al., 2023, 2024; Linde et al., 2024). However, many commercial vegan dog foods do not ensure nutrition adequacy (Dodd et al., 2018). Dodd et al., 2023 and Linde et al., 2024 demonstrated that when fed a plant-based diet, adult dogs maintained health as assessed by physical examinations, complete blood count, serum biochemistry, plasma amino acids, serum vitamins, cardiac biomarkers, serum vitamin D metabolites, and bone mineralization. In one study investigating the fecal microbiome of dogs fed an entirely plant-based diet compared to a commercial animal-based diet with a similar nutrient profile, minimal microbiome changes occurred after 3 mo (Liversidge et al., 2023). Despite, this research, there is still a scarcity of research focusing on how a plant-based diet may impact the fecal metabolome of dogs. The fecal metabolome can aid to provide insight into gut health and act as an indicator of gut microbial activity (Coelho et al., 2018; Karu et al., 2020).

In humans and dogs, long-term dietary habits alter the functional metabolism of the intestinal microbiome, which can expose the host to several beneficial or harmful health factors (Albenberg and Wu, 2014; De Filippis et al., 2016; Lynch and Pedersen, 2016; Schmidt et al. 2018; Alessandri et al., 2019). In human nutrition transitioning to a vegan diet is one of the most extreme human dietary patterns that is sustainable for extended periods of time and shows alterations in the fecal metabolome compared to individuals consuming an omnivorous diet (Wu et al., 2016). Specifically, the fecal metabolome of vegan humans when compared to individuals consuming an omnivorous diet demonstrates increased metabolites associated with carbohydrate fermentation and decreased metabolites associated with protein and fat fermentation (Clarys et al., 2014; David et al., 2014; Wu et al., 2016). The nutrient profile of human plant-based diets; however, is often greatly different from the nutrient profile of omnivorous diets, with plant-based diets typically being lower in protein and fat, and higher in fiber (Thompson, 2008; Tran et al., 2008; Knisken and Johnston, 2011; Clarys et al., 2014).

In contrast to human nutrition, commercial extruded dry diets for dogs, are specifically formulated to meet or exceed standard nutrient recommendations for the intended life stage of the animal (AAFCO, 2019). Thus, regardless of the ingredients selected, the nutrient profile of a plant-based or animal-based diet can be similar in essential nutrient content (Dodd et al., 2023; Liversidge et al., 2023). Although metabolome differences that were noted in human studies are suggested to be due to the difference in nutrient profiles, it remains unclear whether such a shift would occur in dogs fed a plant-based diet, when controlled for nutrient content. Thus, we hypothesized that in healthy adult dogs, the metabolic signature of plant-based diets is distinct from animal-based diets. Specifically, we predicted increases in the concentration of metabolites related to polysaccharide digestion, as seen in humans consuming a vegan diet. Therefore, this study aimed to examine the effects of an experimental extruded plant-based diet compared to a commercial extruded animal-based diet with similar macro- and micronutrient content on the fecal metabolome in dogs.

## Materials and Methods

All experimental procedures for this study were approved by the University of Guelph Animal Care Committee (AUP#4192) and the Research Ethics Board (Research Ethics Approval number 19-02-036), and were in accordance with institutional, provincial, and national guidelines for the care and use of animals and humans participating in research.

### Animals and experimental design

The present study was conducted as part of a larger randomized, double-blinded longitudinal study in client-owned healthy adult dogs, which occurred between July 2019 and November 2020 (Dodd et al., 2023). Details regarding the trial recruitment used were published previously (Dodd et al., 2023; Liversidge et al., 2023, 2024). Recruitment of trial participants was performed by an eSurvey designed on the Qualtrics platform (Provo, Utah, USA) to collect data regarding suitability for study enrollment. The survey was advertised locally around the University of Guelph campus and surrounding community and shared virtually on social media with local dog-related groups. Recruitment resulted in a total of 87 dogs scheduled for enrollment appointments.

The enrollment appointment included a discussion of the study procedures, collection of signed informed consent from participants, and a wellness examination of the dogs conducted by a licensed veterinarian. This wellness examination involved a medical and dietary history and a physical examination. Blood was collected for complete blood count and serum biochemistry. Dogs were approved for inclusion in the trial if they were confirmed to be spayed/neutered, ages 3 yr or older, had a BCS between 4 and 7 on a 9-point-scale (Laflamme, 1997), and were deemed healthy based on a physical examination and routine blood work. Seventy-six dogs met the inclusion criteria after the enrollment appointment and started the 4-wk adaptation period during which all dogs received the same commercial extruded animal-based diet (MEAT). Eleven dogs did not continue the study after the adaptation period due to not eating the diet, gastrointestinal signs, excessive weight gain, or COVID-19 pandemic-related pet guardian dropouts.

The remaining 65 dogs were randomly assigned into 2 diet groups; continuing with the animal-based diet (MEAT, *n* = 31) or being fed an experimental extruded plant-based diet (PLANT, *n* = 34). Diets were fed for 12 wk, maintaining current energy intake, as determined based on diet history information. Four dogs were excluded during the experimental period due to pet guardian personal reasons or dog health concerns unrelated to the diet, including the development of gastrointestinal ulcers after administrations of non-steroidal anti-inflammatory drugs and the development of a urinary tract infection. Fecal collections for fecal metabolomics occurred after 12 wk of exclusively feeding either the MEAT or PLANT diet.

Due to the COVID-19 pandemic and public-health-related restrictions on research involving human participants, the trial was paused for 4 mo from March 2020 until July 2020. During this period, dogs were maintained on the experimental diets, either PLANT or MEAT, depending on which trial phase they were in (adaptation or experimental period), to allow for immediate resumption of data collection when restrictions were lifted, and the study resumed. This resulted in some variation in trial duration for dogs participating in the study, with the adaptation period for 5 dogs lasting more than 4 wk (PLANT *n* = 2; MEAT *n* = 3), the experimental period for 2 dogs lasting more than 12 wk (PLANT *n* = 1; MEAT *n* = 1), and for 3 dogs both the adaption period and the experimental period lasting more than 4 and 12 wk, respectively (PLANT *n* = 2; MEAT *n* = 1). During this period, frequent communication between the research team and the pet guardians was maintained. This communication was performed to aid as a reminder to stay consistent with trial protocol during the extended 4 mo.

### Diets

Both diets, MEAT and PLANT, used in this study were formulated to meet or exceed nutrient recommendations according to the Association of American Feed Control Officials (AAFCO, 2019) nutrient profile for canine adult maintenance (Table 1). The MEAT diet used in this study was a commercial extruded dog food (Petcurean Go! Skin + Coat Care Chicken Recipe, PPN Ltd., Chilliwack, BC, Canada). The PLANT diet was formulated specifically for this trial entirely without animal-derived ingredients to be isoenergetic, isonitrogenous, and as similar in macro-and-micronutrient profiles to the MEAT diet as possible based on AAFCO 2019 recommendations (Table 1). The nutrient profiles and details regarding the ingredients of both diets used were published previously (Dodd et al., 2023; Liversidge et al., 2023, 2024); however, can be found in Table 1. Pet guardians and researchers were blinded to the identity of the diets being fed to study participants throughout the duration of the testing period and remained blinded until after all data was analyzed.

**Table 1.** Nutrient profile on a dry matter basis and complete ingredient list of the experimental vegan (PLANT) and commercial animal-based (MEAT) extruded diets fed to client-owned dogs in this randomized, double-blinded longitudinal study

Nutrient, g/100g DM	PLANT	MEAT
Moisture	6.8	5.9
CP	23.68	27.74
EE	14.9	13.2
CF	3.9	3.4
CA	7.1	8.1
NFE^1^	43.92	41.66
ME^2^ (g/100kcal)	419	410
Total dietary fiber	15.5	9.6
SDF	10.9	2.1
IDF	4.6	7.5
RS	2.7	7.9
Alanine	0.27	0.42
Arginine	0.43	0.48
Aspartic acid	0.64	0.58
Cystine	0.10	0.19
Glutamate	0.80	0.77
Glycine	0.30	0.60
Histidine	0.15	0.15
Isoleucine	0.27	0.23
Leucine	0.50	0.47
Lysine	0.35	0.38
Methionine	0.14	0.18
Methionine + Cystine	0.24	0.37
Phenyalanine	0.36	0.29
Phenyalanine + tyrosine	0.49	0.43
Proline	0.32	0.43
Serine	0.33	0.31
Taurine	0.02	0.06
Threonine	0.26	0.26
Tryptophan	0.03	0.03
Tyrosine	0.13	0.14
Valine	0.33	0.30
**PLANT diet ingredients**		
Peas, barley, oats, potato protein, sunflower oil (preserved with mixed tocopherols), pea protein, lentils, quinoa, calcium carbonate, dicalcium phosphate, primary dried yeast, flaxseed, natural vegetable flavoring, salt, dried marine algae, choline chloride, vitamins (vitamin A supplement, vitamin D2 supplement, vitamin E supplement, niacin, l-ascorbyl-2-polyphosphate (a source of vitamin C), d-calcium pantothenate, thiamin mononitrate, riboflavin, pyridoxine hydrochloride, folic acid, biotin, vitamin B12 supplement), minerals (zinc proteinate, iron proteinate, copper proteinate, zinc oxide, manganese proteinate, copper sulphate, ferrous sulphate, calcium iodate, manganous oxide, selenium yeast), dl-methionine, potassium chloride, l-lysine, taurine, l-carnitine, dried rosemary		
**MEAT diet ingredients**		
Chicken meal, de-boned chicken, whole brown rice, white rice, oatmeal, chicken fat (preserved with mixed tocopherols), potatoes, salmon meal, natural chicken flavor, whole dried egg, flaxseed, pea fiber, alfalfa, apples, carrots, cranberries, sodium chloride, potassium chloride, dried chicory root, dried Lactobacillus acidophilus fermentation product, dried Enterococcus faecium fermentation product, vitamins (vitamin A supplement, vitamin D3 supplement, vitamin E supplement, niacin, l-ascorbyl-2-polyphosphate (a source of vitamin C), d-calcium pantothenate, thiamin mononitrate, beta-carotene, riboflavin, pyridoxine hydrochloride, folic acid, biotin, vitamin B12 supplement), minerals (zinc proteinate, iron proteinate, copper proteinate, zinc oxide, manganese proteinate, copper sulphate, ferrous sulphate, calcium iodate, manganous oxide, selenium yeast), DL-methionine, l-lysine, taurine, yucca schidigera extract, dried rosemary.		

^1^NFE calculated as: 100- CP—EE—CF—CA (AAFCO, 2018).

^2^ME calculated as: 10 × [(3.5 × CP) + (8.5 × EE) + (3.5 × NFE)] (AAFCO, 2019).

DM, dry matter; PLANT, Plant-based diet; MEAT, meat-based diet; CP, crude protein; EE, crude fat; CF, crude fiber; CA; crude ash; NFE, nitrogen-free extract; ME, metabolizable energy; SDF, Soluble dietary fiber; IDF, Insoluble dietary fiber; RS, resistant starch.

Both diets were formulated to be isoenergetic, isonitrogenous, and as similar as possible in nutrient profiles.

Food quantity was calculated based on the dogs’ current dietary intake to match calories and maintain current body weight (BW). A gram scale was provided to each household to precisely measure the recommended quantity of food per day. Pet guardians were given a list of plant-based treats without added micronutrients, and an acceptable treat dose was calculated for each dog to avoid exceeding 10% of their daily energy intake from sources other than the experimental PLANT and MEAT diet. Pet guardians were instructed not to feed their dogs any other food items and to record food and treat intake in a daily food diary for the duration of the study.

### Fecal sample collections

The fecal sample collections occurred at 2 time-points, baseline, and exit. Baseline fecal collections occurred after the 4-wk adaptation period, when all dogs were fed the MEAT diet, and exit fecal collections occurred after 12 wk of exclusively feeding either the MEAT or PLANT diet. Fecal samples were collected immediately after voiding, frozen, and delivered to the researcher in a provided container stored in a Styrofoam cooler box. Samples were weighed and kept in a −20 °C freezer until shipped for analysis.

### Sample preparation

Fecal samples were shipped to The Metabolomic Innovation Center (University of Alberta, Edmonton, AB, Canada) on dry ice for quantitative nuclear magnetic resonance (NMR) spectroscopy. Samples were thawed at room temperature for at least 1-h. Feces were then finely powdered in liquid nitrogen and quickly transferred to an Eppendorf tube. Six hundred microliters of ice-cold high-performance liquid chromatography water were added to the fecal powder (60 to 65 mg) and vortexed vigorously for 5 min. On a shaker, samples were shaken for 25 min at 1,000 rpm at 4 °C, followed by sonication at 4 °C for 15 min. The sonicated sample tubes were centrifuged at 14,000 rpm for 20 min at 4 °C or in the cold room. Supernatant (500 uL) was then transferred to the pre-washed 3 KDa cutoff centrifugal filter units (Amicon Microcon YM-3) and centrifuged at 11,000 rpm for 20 min at 4 °C or in the cold room.

### NMR analysis

A 200 μL filtrate was transferred to an Eppendorf tube and 50 uL buffer (54% D_2_O:46% 1.75 mM KH_2_PO_4_ pH 7.0 v/v containing 5.84 mM DSS (2,2-dimethyl-2-silcepentane-5-sulphonate), 5.84 mM 2-chloropyrimidine-5 carboxylate) was added to it. The sample (250 µL) was then inserted in a 3 mm SampleJet NMR tube for subsequent spectral analysis. All proton NMR (^1^H-NMR) spectra were collected on a 700 MHz Avance III (Bruker) spectrometer equipped with a 5 mm HCN Z-gradient pulsed-field gradient cryoprobe. ^1^H-NMR spectra were acquired at 25 °C using the first transient of the NOESY pre-saturation pulse sequence (noesy1dpr), chosen for its high degree of quantitative accuracy (Saude et al., 2006). All free induction decays were zero-filled to 250K data points. The singlet produced by the sodium trimethylsilyl propanesulfonate (DSS) methyl groups was used as an internal standard for chemical shift referencing (set to 0 ppm) and for quantification all ^1^H-NMR spectra were processed and analyzed using an in-house version of the MAGMET automated analysis software package using a custom metabolite library. MAGMET allows for qualitative and quantitative analysis of an NMR spectrum by automatically fitting spectral signatures from an internal database to the spectrum. Each spectrum was further inspected by an NMR spectroscopist to minimize compound misidentification and misquantification. Typically, all visible peaks were assigned and annotated with a compound name. It has been previously shown that this fitting procedure provides an absolute concentration accuracy of 90% or better (Ravanbakhsh et al., 2015). The metabolites analyzed were categorized into 6 metabolite groups: 1) Amino acids and Amines; 2) Fatty acids; 3) Sugars and sugar metabolites; 4) Alcohols; 5) Nitrogenous bases; and 6) Other metabolites, based on descriptions of each compound available through MetaboAnalyst pathway analysis, the National Library of Medicine PubChem Platforms. Metabolite group classification and compounds analyzed are described in Table 2.

**Table 2.** Metabolite group classifications and compounds of all 66 fecal metabolites that were quantified using nuclear magnetic resonance spectrometry in a randomized, double-blinded longitudinal study

Metabolite group	Compounds
Amino acids and amines	creatine, methylamine, 4-aminobutyrate, betaine, dimethylamine, glycine, isoleucine, taurine, tryptophan, tyrosine, creatinine, phenylacetate, l-carnitine, dimethylglycine, l-glutamic acid, l-phenylalanine, l-alanine, l-proline, l-threonine, l-asparagine, l-histidine, l-lysine, l-serine, l-aspartate, ethanolamine, N6-acetyllysine, l-arginine, l-glutamine, l-leucine, methionine, valine, trimethylamine, trans-4-hydroxy-d-proline, putrescine, cadaverine
Fatty acids	acetic acid, propionate, butyrate, valerate, 3-hydroxyisovaleric acid, isobutyric acid, isovaleric acid, fumaric acid, methylmalonic acid, pyruvic acid, malonate
Sugars and sugar metabolites	d-glucose, d-galactose, l-fucose, succinate, l-lactic acid, xylose, sarcosine, arabinose, fructose,
Alcohols	ethanol, methanol, glycerol, myo-inositol, isopropanol
Nitrogenous bases	uracil, xanthine, hypoxanthine
Other metabolites	choline, acetoin, formate

Fifty-four client-owned dogs of various breeds were exclusively fed either a PLANT (*n* = 30) or MEAT (*n* = 24) diet for 3 mo.

### Statistical analysis

Statistical analysis was performed in R (R Core Team, Vienna, Austria). The distribution of each metabolite and independent variables (dog age, BW, and BCS) was assessed for normality using the Shapiro-Wilk test and visual inspection of frequency histograms. As the data was non-parametric; a mixed model gamma linear regression was employed to evaluate the association of metabolite concentrations with age, sex, BW, and diet. This model was run to keep the data as true to its original form and include diet, BW, sex, and age as fixed effects to assess their influence on metabolite concentrations. To examine interactions between diet and time, comparisons were made between the PLANT and the MEAT group at each time-point (baseline and exit) and between time-points within diet groups (baseline-exit). These correlations were evaluated again using mixed model gamma linear regression with age, sex, and BW included as covariates. As multiple comparisons were made, the critical cutoff for *P*-values was adjusted to control for inflated type I error using Tukey’s honest significant difference. A *P* < 0.05, adjusted using Tukey’s honest significant difference, for all comparisons was considered statistically significant.

Data with significant associations detected with mixed model gamma linear regression were uploaded to the Metaboanalyst 5.0 platform and normalized for comparison by row-wise normalization (median or quantile normalization), mathematical transformation (log or square root), and/or data scaling (autoscaling) as appropriate. Visual inspection of density plots and box plots was performed to evaluate the normalization technique. The overview of data was visualized using interactive principal component analysis and 2-way heatmaps were generated with Euclidean distance measure and Ward clustering algorithm.

## Results

All 61 dogs completing the study remained in good health; however, the sample size was reduced to 54 (PLANT *n* = 30; MEAT *n* = 24) for statistical analysis due to the absence of fecal samples provided by some pet guardians at either baseline or exit collection. The demographic data, including breed, age, BW, BCS, and sex of the 54 dogs included in statistical analysis are presented in Supplementary Table 1.

As mentioned previously, the COVID-19 pandemic added variation in trial duration for 10 dogs participating in the study. Examination of labeled scatter plots revealed these dogs were not identified as outliers and results were not significantly different from the dogs consuming the experimental diet for the intended 12 wk.

Through NMR spectroscopy, 66 metabolites were quantified from all fecal samples: 35 amino acids and amines; 10 fatty acids; 9 sugars and sugar metabolites; 5 alcohols; 3 nitrogenous bases; and 4 other metabolites (Table 2).

Mixed model gamma linear regression showed that age, BW, sex, and diet over time were significantly associated with 49 out of 66 metabolites fecal metabolite concentrations (Supplemental Table 2). Increasing age was associated with higher concentrations of 5 out of 49 metabolites (2 amino acids, 2 fatty acids, and 1 sugar and other sugar metabolites). Additionally, Increasing BW was associated with higher metabolite concentrations of 21 of the 49 metabolites (13 amino acids, 4 fatty acids, 3 sugar and other sugar metabolites, and one alcohol). Increasing both age and BW presented a pattern that an increase in metabolite concentration would occur for every increase in age or BW. Sex had a significant association with 6 of the 49 metabolites (2 amino acids, 2 fatty acids, and 2 sugar and other sugar metabolites), with males demonstrating higher metabolite concentrations compared to females. Interactions between diet and time were also present in 17 out of 66 metabolites (3 amino acids, 5 fatty acids, 6 sugar and other sugar metabolites, 2o alcohols, 1 nitrogenous base).

**Figure 1.**
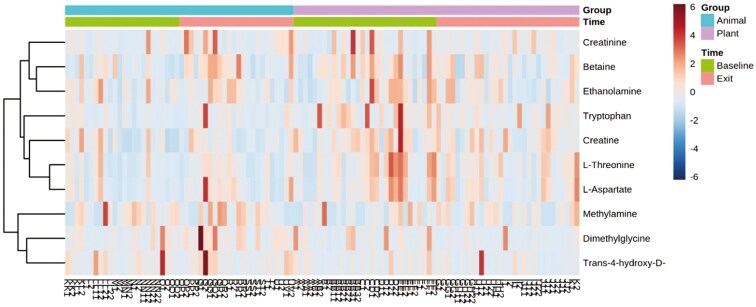
Two-way heatmap of significant amino acid and amine metabolites in 54 client-owned healthy adult dogs (*n* = 25 neutered male, and *n* = 29 spayed female) participating in a randomized, double-blinded longitudinal study. Dogs were exclusively fed either a PLANT (*n* = 30) or MEAT (*n* = 24) diet for 3 mo.

**Figure 2.**
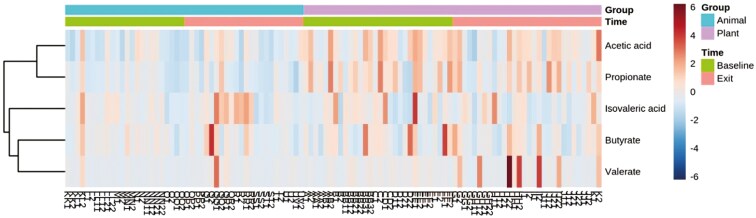
Two-way heatmap of significant fatty acid metabolites in 54 client-owned healthy adult dogs (*n* = 25 neutered male, and *n* = 29 spayed female) participating in a randomized, double-blinded longitudinal study. Dogs were exclusively fed either a PLANT (*n* = 30) or MEAT (*n* = 24) diet for 3 mo.

**Figure 3.**
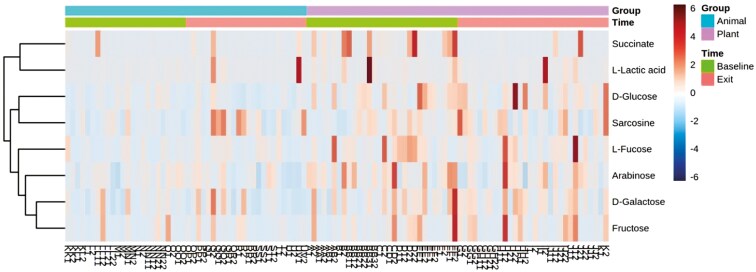
Two-way heatmap of significant sugars and other sugar metabolites in 54 client-owned healthy adult dogs (*n* = 25 neutered male, and *n* = 29 spayed female) participating in a randomized, double-blinded longitudinal study. Dogs were exclusively fed either a PLANT (*n* = 30) or MEAT (*n* = 24) diet for 3 mo.

When controlling for age, BW, and sex, 43 out of 66 metabolites showed significant changes over time in response to switching from the MEAT diet to the PLANT diet. These changes included 13 amino acids and amines (Figure 1, Supplementary Table 3), 8 fatty acids (Figure 2, Supplementary Table 4), 14 sugars and sugar metabolites (Figure 3, Supplementary Table 5), 4 alcohols (Supplementary Figure 1 and Table 6), 4 nitrogenous base and other metabolites (Supplementary Fig 2 and Table 7).

Specifically, at the exit time-point when comparing the PLANT and MEAT diet groups, 20 out of 43 significant metabolites showed significant changes over time. Higher concentrations at the exit time-point were seen in 17 out of the 20 metabolites (3 amino acids, 3 fatty acids, 7 sugars, and other sugar metabolites, 2 alcohols, 2 nitrogenous, and other metabolites) when compared to the MEAT group. Whereas, the remaining 3 out of the 20 metabolites (2 amino acids and 1 fatty acids) were seen to be higher in the MEAT group at exit time-point. These results are presented in Figures 1 to 3, Supplementary Tables 3 to 7 and Figures 1 to 2. Comparisons within diet groups over time (baseline-exit) demonstrated that 21 out of 43 metabolites in the PLANT group and 2 out of 43 metabolites in the MEAT group showed significant changes over time. Specifically in the PLANT group, 12 out of 21 metabolites (2 amino acids, 2 fatty acids, 7 sugars, and 1 alcohol) increased over time. Whereas 9 out of 21 metabolites (5 amino acids, 1 fatty acid, 1 alcohol, and 2 nitrogenous base and other metabolites) decreased in the PLANT group over time. These results can be found in Figures 1 to 3, Supplementary Tables 3 to 7, and Figures 1 to 2. In the MEAT group over time, 2 of the 43 metabolites (1 amino acid and 1 fatty acid) decreased over time. These results can be found in Figures 1 to 2 and Supplementary Tables 3 and 4.

## Discussion

The present study investigated the effects of an experimental plant-based diet on the fecal metabolome of healthy adult pet dogs using NMR spectroscopy. As hypothesized, the study identified a distinct fecal metabolomic signature of dogs after shifting their diet from a meat-based to an entirely plant-based diet. More specifically, 46 out of 66 fecal metabolites increased over time in response to the PLANT diet. Differences in metabolite concentrations were observed both over time within groups and between the PLANT and MEAT groups at specific time points. In the current study, the increase in fecal sugar metabolites supported the hypothesis that the plant-based diet would prompt carbohydrate fermentation. Similar patterns have been reported in human studies, where higher fecal sugar metabolites are associated with increased carbohydrate intake and fermentation in individuals consuming a vegan diet (Clarys et al., 2014; David et al., 2014; Wu et al., 2016).

In humans, long-term dietary habits have a large impact on the gut microbiota and corresponding fecal metabolites (Wu et al., 2014; Neis et al., 2015; Galie et al., 2021). A 2-way relationship exists between the microbiota and metabolome of the gut (Nguyen et al., 2021), where nutrients consumed by the host shape the composition of the gut microbiota, and the gut microbiota aid in regulating the host metabolic homeostasis by supporting the fermentation of nutrients (Fiehn et al., 2000; Dai, 2011; De Brito et al., 2021; Nguyen et al., 2021). In humans, dietary habits, particularly between omnivorous and vegan diets, significantly impact the gut microbiota and corresponding metabolomic profile (Wu et al., 2011; Davila et al., 2013; Deng and Swanson, 2015; Li et al., 2017; Wishart, 2019; Nguyen et al., 2021). Specifically, in general, the fecal microbiota in humans is characterized by the dominance of Bacteroidetes and Firmicutes phyla (Simpson and Campbell, 2015; Bamberger et al., 2018). Research demonstrated the increased relative abundance of Bacteroidetes with consumption of vegan diets compared to individuals consuming an omnivorous diet (Losasso et al., 2018; Jain et al., 2018). The authors previously reported that in the current study, the canine fecal microbiota response to the plant-based diet was distinct from reports in human literature (Liversidge et al., 2024). Dogs consuming the plant-based diet had lower Bacteroidetes abundance compared to those consuming the animal-based diet. Despite these minimal and distinct changes from what is reported in human literature, the dogs in the current study still expressed notable metabolite shifts towards carbohydrate fermentation when consuming the PLANT diet compared to the MEAT diet. These conflicting results may be due to the many exogenous factors affecting the microbiota within the gastrointestinal tract (Swanson et al., 2001; Wu et al., 2011; Li et al., 2017; Liversidge et al., 2024). Specifically, the metabolite shift described in human literature may be a result of different nutrient profiles of vegan versus omnivorous diets, with vegan diets being lower in total energy and protein intake but increased in fat and fiber intake when compared to omnivorous diets (Dodd et al., 2018; Liversidge et al., 2023; Clarys et al., 2024). These changes beg the question of whether the changes in human fecal microbiota and metabolome may be in response to nutrient differences in the diets, not in response to different ingredients themselves.

In comparison to human vegan diets, commercial plant-based diets for dogs are designed to meet industry guidelines for dogs and are specifically formulated to meet or exceed standard nutrient recommendations (Tanprasertsuk et al., 2021; Liversidge et al., 2023). Despite the ingredients used, a plant-based diet formulated to meet or exceed nutrient recommendations for adult maintenance and exposed to similar processing methods as conventional animal-based pet foods may be a suitable alternative diet for dogs (Dodd et al., 2018; Liversidge et al., 2023). Recent studies demonstrated that plant-based diets have similar apparent total-tract nutrient digestibility and fecal microbiota profiles when compared to commercial animal-based diets (Linde et al., 2024 and; Liversidge et al., 2023, 2024). Furthermore, dogs fed a well-balanced entirely plant-based diet maintained overall health as noted by assessment of serum total vitamin D concentrations, bone mineralization, physical exams, complete blood count, and serum biochemistry (Dodd et al., 2023; Linde et al., 2024). The evidence supporting plant-based diets heavily recognizes it is more nutrients that drive impact on gastrointestinal health impact then ingredients which places large responsibility on manufacturing and processing of plant-based diets.

Although the diet was designed to be isoenergetic and as similar in macro- and micronutrient profile as possible to the MEAT diet, post-manufacturing analyses revealed slight nutrient variations. These differences may explain the enhanced carbohydrate fermentation observed with the PLANT diet in the current study. The PLANT diet contained barley, oats, quinoa, flaxseed, and primary dried yeast whereas the MEAT diet contained brown and white rice, oatmeal, flaxseed, pea fiber, and alfalfa. This likely affected dietary fiber content. Note that based on AAFCO 2019 recommendations, crude fiber (CF) was included as a formulation parameter, but other carbohydrate and fiber measurements such as soluble dietary fiber (SDF), insoluble dietary fiber (IDF), and resistant starch (RS), were not accounted for in formulation. Post-manufacturing analysis of CF, calculated nitrogen-free extract (NFE), SDF, IDF, and RS found slightly different levels between the diets. Specifically, the PLANT diet had higher levels of CF, NFE, SDF, and IDF, but lower levels of RS compared to the MEAT diet. In both humans and dogs, we know that if the host consumes a diet high in protein and low in carbohydrates, the colonic environment will shift to harbor more bacteria that aid in proteolytic fermentation and vice versus high carbohydrates will shift to harbor more bacteria needed for carbohydrate fermentation (Macfarlane and Macfarlane, 2003; Middelbos et al., 2007; Morowitz et al., 2011; Maier et al., 2017; Eisenhauer et al., 2019; Jackson et al., 2020; Liu et al., 2021). Thus, while formulation targets may be set during diet design, actual nutrient composition can shift during production which can affect the way nutrients are processed in the gastrointestinal tract.

The authors note that the current study was not without limitations. The use of client-owned dogs, which necessitates guardian compliance could be viewed as a study limitation. The increased metabolites related to polysaccharide digestion detected in the PLANT group, suggest that participants complied with the dietary instructions and refrained from feeding animal products to their dogs for the duration of the study protocol. Furthermore, as previously mentioned this trial was part of a larger study including investigations on vitamin D and evaluation of guardian compliance during in-home digestibility trials (Dodd et al., 2023; Liversidge et al., 2024). Dodd et al. (2023) reported dogs fed the PLANT diet had higher circulating vitamin D_2_ concentrations which provided a good indication that the dogs consumed the PLANT diet throughout the study. During the apparent total-tract nutrient digestibility study, the presence or absence of guardian-reported food diary information was reported to show no effect on the overall results (Liversidge et al., 2024). For these reasons, this limitation did not interfere with the study findings. Further, the diet can be seen as a limitation. As discussed above, slight differences occurred between the formulated nutrient profile and the analyzed nutrient profile post-manufacturing for both diets, which may have affected the study outcomes. Moreover, only a single plant-based diet was tested. Different formulations of plant-based diets could have varying impacts on the fecal metabolome and overall health, and the results cannot be generalized to all plant-based diets.

## Conclusion

The current study found that the fecal metabolic signature of healthy adult dogs fed an experimental extruded entirely plant-based (vegan) diet was distinct from dogs consuming a traditional animal-based diet. These results may have been due to slight nutrient profile differences between the PLANT and MEAT diets. It is noteworthy that the results of this study are specific to the diets used in this study. Given the rising popularity of plant-based diets for dogs, there is a need for future research to take into consideration plant-based diets with various nutrient profiles to gain an understanding of how various formulations could impact the fecal metabolome.

## Supplementary Material

skaf054_suppl_Supplementary_Figures_1-2_Tables_1-7

## Abbreviations

BW: body weight
BCS: body condition score
AAFCO: The Association of American Feed Control Officials
NMR: nuclear magnetic resonance
HPLC: high performance liquid chromatography
^1^H-NMR: proton nuclear magnetic resonance
SCFA: short-chained fatty acids
DM: dry matter
NFE: nitrogen-free extract
SDF: soluble digestible fiber
IDF: indigestible fiber
