# Supplementary material for: The fecal metabolomic signature of a plant-based (vegan) diet compared to an animal-based diet in healthy adult client-owned dogs
Source: J Anim Sci. 2025 Feb 27;103:skaf054. doi: 10.1093/jas/skaf054 (PMC12056932; doi:10.1093/jas/skaf054)
Supplement: skaf054_suppl_Supplementary_Figures_1-2_Tables_1-7 [file skaf054_suppl_supplementary_figures_1-2_tables_1-7.zip › Supplemental Table 6_Alcohol.docx]

Table S6. Alcohol metabolite concentrations quantified from the feces of 54 client-owned healthy adult dogs (n=25 neutered male, and n= 29 spayed female) participating in a randomized, double-blinded longitudinal study. Dogs were exclusively fed either a PLANT (n=30) or MEAT (n=24) diet for 3 months.

| **Metabolite** | **PLANT^1^**  **Baseline** | **PLANT^1^**  **Exit** | **MEAT^2^**  **Baseline** | **MEAT^2^**  **Exit** | **Association of PLANT^1^ over time**  **P-value** | **Association of MEAT^2^ over time**  **p-value** | **Association of PLANT^1^ vs MEAT^2^ Baseline**  **p-value** | **Association of PLANT^1^ vs MEAT^2^ Exit**  **p-value** |
| --- | --- | --- | --- | --- | --- | --- | --- | --- |
| Ethanol | 2.88  (0.63-14.97) | 5.07  (1.57-17.42) | 2.22  (0.72-4.74) | 3.75  (0.76-27.32) | <0.0001^b^ | 0.40 | 0.16 | <0.0001^c^ |
| Methanol | 4.78  (2.10-10.23) | 4.53  (2.71-8.58) | 4.70  (2.84-14.86) | 4.31  (2.67-8.76) | 0.29 | 0.61 | 1.00 | <0.001^c^ |
| Glycerol | 14.18  (6.95-21.49) | 12.00  (4.44-24.44 | 14.74  (8.80-18.71) | 12.50  (6.98-20.34) | 0.20 | 0.10 | 0.40 | 0.20 |
| Myo-inositol | 1.37  (0.15-3.61) | 1.65  (0.08-3.73) | 1.06  (0.15-3.26) | 1.31  (0.03-3.65) | 0.32 | 1.00 | 0.84 | 0.13 |
| Isopropanol | 0.25  (0.06-0.77) | 0.20  (0.02-0.91) | 0.20  (0.09-0.44) | 0.15  (0.05-0.44) | 0.01^a^ | 0.64 | 0.22 | 0.84 |

Evaluation of interactions between diet and time were made between the two diet groups per timepoint and between two time-points within diet groups using mixed model gamma linear regression controlling for age, sex, and BW.
Data was presented as non-parametric metabolite concentrations between diet group at each timepoint are presented as median and interquartile range [minimum and maximum]. ^1^PLANT= plant-based diet
^2^MEAT=animal-based diet
^a^Denotes significant decrease in metabolite concentration over time
^b^Denotes a significant increase in metabolite concentration over time
^c^Denotes higher concentration in the PLANT group compared to the MEAT group
