# Supplementary material for: The fecal metabolomic signature of a plant-based (vegan) diet compared to an animal-based diet in healthy adult client-owned dogs
Source: J Anim Sci. 2025 Feb 27;103:skaf054. doi: 10.1093/jas/skaf054 (PMC12056932; doi:10.1093/jas/skaf054)
Supplement: skaf054_suppl_Supplementary_Figures_1-2_Tables_1-7 [file skaf054_suppl_supplementary_figures_1-2_tables_1-7.zip › Supplemental T2_GLR Association.docx]

Table S2 Mix effect model using gamma linear regression evaluating the association between age, sex, and body weight quantified from the feces of 54 client-owned healthy adult dogs of various breed participating in a randomized, double-blinded longitudinal study. Dogs were exclusively fed either a PLANT (n=30) or MEAT (n=24) diet for 3 months.

| **Metabolite** | **Association of Age p-value** | **Association of Sex p-value** | **Association of BW^1^ p-value** |
| --- | --- | --- | --- |
| **Amino acid and amines** | | | |
| Creatine | 0.94 | 0.17 | 0.33 |
| Methylamine | 0.40 | 0.45 | 0.39 |
| 4-aminobutyrate | 0.71 | 0.48 | 0.68 |
| Betaine | 0.60 | 0.18 | 0.61 |
| Dimethylamine | 0.88 | 0.99 | 0.60 |
| Glycine | 0.40 | 0.28 | 0.01^a^ |
| Isoleucine | 0.96 | 0.30 | 0.03^a^ |
| Taurine | 0.05 | 0.80 | 0.21 |
| Tryptophan | 0.11 | 0.79 | 0.97 |
| Tyrosine | 0.52 | 0.12 | <0.001^a^ |
| Creatinine | 0.30 | 0.98 | 0.86 |
| Phenylacetate | 0.05 | 0.75 | 0.19 |
| L-carnitine | 0.57 | 0.36 | 0.15 |
| Dimethylglycine | 0.05 | 0.97 | 0.73 |
| L-glutamic acid | 0.58 | 0.31 | <0.0001^a^ |
| L-phenylalanine | 0.82 | 0.16 | 0.14 |
| L-alanine | 0.34 | 0.54 | 0.04^a^ |
| L-proline | 0.42 | 0.67 | <0.001^a^ |
| L-Threonine | 0.40 | 0.31 | <0.001^a^ |
| L-asparagine | 0.26 | 0.72 | 0.04^a^ |
| L-histidine | 0.68 | 0.96 | 0.99 |
| L-lysine | 0.09 | 0.13 | <0.0001^a^ |
| L-serine | 0.45 | 0.22 | 0.03^I^ |
| L-aspartate | 0.60 | 0.12 | 0.06 |
| Ethanolamine | <0.001^a^ | <0.001^a^ | <0.001^a^ |
| N6-acetyllysine | 0.33 | 0.74 | 0.01^a^ |
| L-arginine | 0.44 | 0.07 | 0.05 |
| L-glutamine | 0.02^a^ | 0.76 | 0.03^a^ |
| L-leucine | 0.13 | 0.19 | 0.02^a^ |
| Methionine | 0.38 | 0.24 | <0.001^a^ |
| Valine | 0.58 | 0.27 | 0.01^a^ |
| Trimethylamine | 0.41 | 0.26 | 0.25 |
| Trans-4-hydroxy-D-proline | 0.46 | 0.03^a^ | 0.10 |
| Putrescine | 0.35 | 0.88 | 0.22 |
| Cadaverine | 0.63 | 0.35 | 0.73 |
| **Fatty acids metabolites** | | | |
| Acetic acid | 0.70 | 0.37 | 0.01^a^ |
| Propionate | 0.08 | 0.28 | 0.13 |
| Butyrate | <0.001^a^ | <0.001^a^ | <0.001^I^ |
| Valerate | 0.66 | 0.01^a^ | 0.25 |
| 3-hydroxyisovaleric acid | 0.33 | 0.42 | <0.001^a^ |
| Isobutyric acid | 0.12 | 0.76 | 0.45 |
| Isovaleric acid | 0.18 | 0.81 | 0.28 |
| Fumaric acid | 0.53 | 0.10 | 0.31 |
| Methylmalonic acid | 0.04^a^ | 0.48 | 0.05 |
| Pyruvic acid | 0.34 | 0.55 | <0.001^a^ |
| Malonate | 0.37 | 0.30 | 0.39 |
| **Sugars and sugar metabolites** | | | |
| D-glucose | 0.36 | 0.29 | 0.55 |
| D-galactose | 0.02^a^ | 0.16 | <0.0001^a^ |
| L-fucose | 0.72 | 0.65 | 0.01^a^ |
| Succinate | 0.13 | <0.001^a^ | 0.65 |
| L-lactic acid | 0.17 | 0.40 | 0.23 |
| Xylose | 0.48 | 0.64 | 0.70 |
| Sarcosine | 0.06 | 0.49 | 0.85 |
| Arabinose | 0.14 | <0.0001^I^ | 0.22 |
| Fructose | 0.72 | 0.65 | 0.01^a^ |
| **Alcohols** | | | |
| Ethanol | 0.44 | 0.80 | 0.65 |
| Methanol | 0.98 | 0.90 | 0.72 |
| Glycerol | 0.20 | 0.39 | 0.74 |
| Myo-inositol | 0.07 | 0.15 | <0.001^a^ |
| Isopropanol | 0.06 | 0.42 | 0.57 |
| **Nitrogenous bases and other metabolites** | | | |
| Uracil | 0.19 | 0.81 | 0.55 |
| Xanthine | 0.21 | 0.75 | 0.24 |
| Hypoxanthine | 0.43 | 0.36 | 0.11 |
| Choline | 0.93 | 0.24 | 0.73 |
| Acetoin | 0.30 | 0.38 | 0.33 |
| Formate | 0.74 | 0.25 | 0.11 |

^1^BW, body weight
^2^Significant p-value accepted as >0.05
^a^Denoting significant association on metabolite concentration.
