# Supplementary material for: The fecal metabolomic signature of a plant-based (vegan) diet compared to an animal-based diet in healthy adult client-owned dogs
Source: J Anim Sci. 2025 Feb 27;103:skaf054. doi: 10.1093/jas/skaf054 (PMC12056932; doi:10.1093/jas/skaf054)
Supplement: skaf054_suppl_Supplementary_Figures_1-2_Tables_1-7 [file skaf054_suppl_supplementary_figures_1-2_tables_1-7.zip › Supplemental Table 3_AA.docx]

Table S3. Amino acids and anime metabolite concentrations quantified from the feces of 54 healthy adult dogs [n=25 neutered male, and n= 29 spayed female] participating in a randomized, double-blinded longitudinal study. Dogs were exclusively fed either a PLANT [n=30] or MEAT [n=24] diet for 3 months.

| **Metabolite** | **PLANT^1^ Baseline** | **PLANT^1^ Exit** | **MEAT^2^**  **Baseline** | **MEAT^2^**  **Exit** | **Association of PLANT^1^**  **Overtime**  **P-value** | **Association of MEAT^2^**  **Overtime**  **p-value** | **Association of PLANT^1^ vs MEAT^2^**  **Baseline**  **p-value** | **Association of PLANT^1^ vs MEAT^2^**  **Exit**  **p-value** |
| --- | --- | --- | --- | --- | --- | --- | --- | --- |
| Creatine | 0.87  [0.12-3.49] | 0.94  [0.18-1.78] | 0.71  [0.10-1.69] | 0.50  [0.00-2.09] | 0.97 | 0.66 | 0.24 | 0.01^c^ |
| Methylamine | 0.67  [0.03-2.17] | 0.35  [0.02-1.79] | 0.74  [0.03-2.52] | 0.40  [0.03-1.61] | <0.001^a^ | 0.50 | 1.00 | 0.43 |
| 4-aminobutyrate | 0.33  [0.07-3.53] | 0.48  [0.00-1.65] | 0.22  [0.03-1.29] | 0.39  [0.08-3.76] | 0.96 | 0.87 | 0.54 | 0.72 |
| Betaine | 0.45  [0.14-0.87] | 0.34  [0.14–0.91] | 0.28  [0.14-0.91] | 0.30  [0.14-0.79] | <0.001^a^ | 0.90 | 0.86 | 0.95 |
| Dimethylamine | 0.08  [0.01-0.44] | 0.07  [0.04-0.24] | 0.08  [0.03-0.24] | 0.08  [0.03-0.38] | 1.00 | 0.97 | 0.89 | 0.76 |
| Glycine | 8.98  [2.44-26.02] | 9.59  [3.68-17.46] | 7.84  [3.99-17.20] | 6.55  [2.39-46.10] | 0.99 | 0.97 | 0.64 | 0.41 |
| Isoleucine | 4.23  [0.81-21.17] | 5.73  [1.82-16.69] | 3.73  [0.96-12.76] | 3.78  [1.50-13.88] | 0.39 | 0.90 | 0.99 | 0.91 |
| Taurine | 7.20  [0.69-37.85] | 5.92  [0.17-34.29] | 6.64  [0.47-11.93] | 2.05  [0.29-11.92] | 0.88 | 0.40 | 0.42 | 0.16 |
| Tryptophan | 0.32  [0.12-2.05] | 0.37  [0.10-1.68] | 0.26  [0.04-0.73] | 0.26  [0.04-1.83] | 0.34 | 0.95 | 0.58 | <0.001^c^ |
| Tyrosine | 3.88  [0.99-17.14] | 5.36  [0.00 – 12.41 | 3.03  [0.14-7.89] | 2.87  [0.70-10.07] | 0.95 | 0.34 | 0.08 | 0.67 |
| Creatinine | 1.30  [0.19-5.85] | 0.70  [0.21-1.77] | 0.84  [0.29-2.64] | 0.56  [0.15-4.92] | <0.001^a^ | 0.74 | 0.14 | 0.18 |
| Phenylacetate | 1.01  [0.19-1.83] | 1.22  [0.10-341] | 0.92  [0.07-3.41] | 0.85  [0.18-2.31] | 0.50 | 0.80 | 0.95 | 0.08 |
| L-carnitine | 0.27  [0.06-0.71] | 0.30  [0.11-0.66] | 0.26  [0.10-0.57] | 0.24  [0.09-0.53] | 0.99 | 0.12 | 1.00 | 0.26 |
| Dimethylglycine | 0.12  [0.04-0.86] | 0.09  [0.04-0.82] | 0.14  [0.04-1.77] | 0.10  [0.03-0.92] | 0.02^a^ | 0.59 | 0.80 | 0.20 |
| L-glutamic acid | 11.40  [2.76-33.09] | 13.04  [5.06-46.02] | 8.65  [2.76-24.39] | 9.37  [2.81-25.89] | 0.32 | 0.83 | 0.80 | 0.11 |
| L-phenylalanine | 3.01  [0.17-12.49] | 3.64  [0.74-8.57] | 2.34 [0.74±7.14] | 2.46  [1.33-6.97] | 0.56 | 1.00 | 0.70 | 0.34 |
| L-alanine | 12.08  [4.34-42.86] | 14.93  [2.88-26.50] | 9.89  [5.74-25.44] | 10.25  [3.96-37.82] | 0.93 | 0.93 | 0.26 | 0.29 |
| L-proline | 3.23  [0.49-12.11] | 3.56  [0.92-7.27] | 2.89  [0.53-7.27] | 2.87  [0.88-8.11] | 0.87 | 0.98 | 0.96 | 0.99 |
| L-Threonine | 3.65  [1.10-15.16] | 5.92  [1.56-12.83] | 3.05  [1.60-7.07] | 2.75  [1.30-10.05] | 0.01^b^ | 0.88 | 0.60 | <0.001^c^ |
| L-asparagine | 1.49  [0.25-8.23] | 1.79  [0.38-7.07] | 0.98  [0.20-7.07] | 1.06  [0.19-8.43] | 0.96 | 1.00 | 0.65 | 0.52 |
| L-histidine | 0.28  [0.06-4.40] | 1.78  [0.07-3.25] | 0.28  [0.06-2.86] | 0.25  [0.10-3.21] | 0.61 | 0.86 | 0.96 | 0.05 |
| L-lysine | 6.18.  [0.07-19.97] | 7.47  [0.84-26.52] | 6.24  [0.00-14.90] | 5.83  [2.91-23.92] | 1.00 | 0.91 | 0.96 | 1.00 |
| L-serine | 5.11  [0.18-19.32] | 6.18  [2.39-13.94] | 3.93  [1.77-10.90] | 4.30  [1.61-13.08] | 0.21 | 1.00 | 0.79 | 0.25 |
| L-aspartate | 4.35  [1.64-21.52] | 8.29  [2.10-18.31] | 3.93  [1.64-10.87] | 3.85  [1.30-28.49] | <0.001^b^ | 0.92 | 0.95 | 0.35 |
| Ethanolamine | 2.57  [0.70-9.59] | 3.02  [0.24-6.27] | 2.12  [0.70-6.27] | 2.26  [0.46-7.08] | <0.0001^a^ | <0.0001^a^ | <0.0001^c^ | <0.0001^c^ |
| N6-acetyllysine | 0.42  [0.05-1.85] | 0.62  [0.22-1.48] | 0.46  [0.05-1.10] | 0.44  [0.07-1.08] | 0.70 | 0.62 | 0.41 | 1.00 |
| L-arginine | 2.09  [0.84-4.82] | 1.73  [0.42-4.31] | 1.83  [0.79-4.40] | 1.35  [0.58-3.73] | 0.81 | 0.50 | 0.14 | 0.05 |
| L-glutamine | 2.40  [0.78-7.29] | 2.34  [0.66-5.21] | 1.58  [0.78-4.76] | 2.01  [0.63-3.75] | 0.86 | 0.65 | 0.97 | 0.85 |
| L-leucine | 7.08  [1.90-27.86] | 10.05  [3.85-25.50] | 6.60  [3.26-17.17] | 6.05  [3.28-17.25] | 0.23 | 0.99 | 0.96 | 0.45 |
| Methionine | 2.65  [0.08-10.92] | 4.16  [0.87-8.05] | 2.23  [1.00-7.27] | 2.07  [0.60-7.28] | 0.75 | 1.00 | 0.79 | 0.38 |
| Valine | 7.73  [1.62-30.19] | 10.85  [3.52-24.86] | 6.60  [2.73-16.10] | 5.23  [2.51-18.35] | 0.54 | 1.00 | 0.93 | 0.62 |
| Trimethylamine | 0.39  [0.10-1.22] | 0.37  [0.19- 0.64] | 0.39  [0.20-0.62] | 0.32  [0.09-0.82] | 0.72 | 0.27 | 0.97 | 0.64 |
| Trans-4-hydroxy-D-proline | 0.23  [0.00-0.78] | 0.06  [0.00-1.77] | 0.22  [0.00-1.87] | 0.16  [0.03-2.41] | 0.04^a^ | 0.89 | 0.71 | 0.02^d^ |
| Putrescine | 1.18  [0.03-5.59] | 1.24  [0.33-3.68] | 0.97  [0.00-3.68] | 1.28  [0.01-2.88] | 0.97 | 0.73 | 0.94 | 0.61 |
| Cadaverine | 3.71  [0.94-20.13] | 3.92  [0.68-9.12] | 3.69  [1.36-9.12] | 3.19  [0.69-15.89] | 0.92 | 0.95 | 0.97 | 0.97 |

Evaluation of interactions between diet and time were made between the two diet groups per timepoint and between two time-points within diet groups using mixed model gamma linear regression controlling for age, sex, and BW.
Data was presented as non-parametric metabolite concentrations between diet group at each timepoint are presented as median and interquartile range [minimum and maximum]. ^1^PLANT= plant-based diet
^2^MEAT=animal-based diet
^a^Denotes significant decrease in metabolite concentration over time
^b^Denotes a significant increase in metabolite concentration over time
^c^Denotes higher concentration in the PLANT group compared to the MEAT group
^d^Denotes lower concentration in the PLANT group compared to the MEAT group
