# Supplementary material for: The fecal metabolomic signature of a plant-based (vegan) diet compared to an animal-based diet in healthy adult client-owned dogs
Source: J Anim Sci. 2025 Feb 27;103:skaf054. doi: 10.1093/jas/skaf054 (PMC12056932; doi:10.1093/jas/skaf054)
Supplement: skaf054_suppl_Supplementary_Figures_1-2_Tables_1-7 [file skaf054_suppl_supplementary_figures_1-2_tables_1-7.zip › Supplemental Table 5_Sugars.docx]

Table S5. Sugars and sugar metabolite concentrations quantified from the feces of 54 client-owned healthy adult dogs (n=25 neutered male, and n= 29 spayed female) participating in a randomized, double-blinded longitudinal study. Dogs were exclusively fed either a PLANT (n=30) or MEAT (n=24) diet for 3 months.

| **Metabolite** | **PLANT^1^ Baseline** | **PLANT^1^ Exit** | **MEAT^2^**  **Baseline** | **MEAT^2^**  **Exit** | **Association of PLANT^1^ over time**  **P-value** | **Association of MEAT^2^**  **over time**  **p-value** | **Association of PLANT^1^ vs MEAT^2^**  **Baseline**  **p-value** | **Association of PLANT^1^ vs MEAT^2^**  **Exit**  **p-value** |
| --- | --- | --- | --- | --- | --- | --- | --- | --- |
| D-glucose | 9.22  (4.16-55.17) | 12.92  (3.58-38.43) | 10.19  (4.16-18.28) | 8.48  (2.75-23.12) | <0.0001^a^ | 0.22 | 0.34 | <0.001^b^ |
| D-galactose | 4.71  (1.64-14.08) | 6.19  (2.03-26.34) | 5.35  (1.64-13.39) | 4.94  (1.32-19.96) | <0.0001^a^ | 0.95 | 0.78 | <0.0001^b^ |
| L-fucose | 0.70  (0.06-5.12) | 1.60  (0.01-10.74) | 0.49  (0.06-2.89) | 0.62  (0.04-1.62) | 0.04^a^ | 1.00 | 0.57 | <0.001^b^ |
| Succinate | 0.24  (0.02-90.44) | 2.11  (0.03-111.99) | 0.21  (0.02-63.19) | 0.14  (0.03-50.72) | 0.03^a^ | 0.82 | 0.52 | <0.001^b^ |
| L-lactic acid | 2.08  (0.76-102.94) | 2.55  (1.15-81.85) | 1.64  (0.89-81.85) | 1.58  (0.75-45.84) | 0.07 | 0.94 | 1.00 | 0.02^b^ |
| Xylose | 6.34  (0.04-16.95) | 3.64  (0.29-15.92) | 7.43  (0.72-16.74) | 3.35  (0.00-12.11) | 0.61 | 0.41 | 1.00 | 0.94 |
| Sarcosine | 0.07  (0.02-0.31) | 0.09  (0.04-0.26) | 0.06  (0.02-0.26) | 0.07  (0.02-0.28) | 0.02^a^ | 0.51 | 0.99 | 0.20 |
| Arabinose | 9.00  (3.73-25.74) | 10.55  (1.87-60.84) | 10.01  (2.13-16.32) | 8.62  (3.56-16.10) | <0.0001^a^ | 1.00 | 0.69 | <0.001^b^ |
| Fructose | 1.05  (0.26-9.64) | 2.00  (0.40-17.22) | 1.08  (0.45-9.55) | 1.15  (0.33-8.88) | 0.04^a^ | 1.00 | 0.57 | <0.001^b^ |

Evaluation of interactions between diet and time were made between the two diet groups per timepoint and between two time-points within diet groups using mixed model gamma linear regression control for age, sex, and BW.
As data was presented as non-parametric metabolite concentrations between diet group at each timepoint are presented as Median and interquartile range (minimum and maximum).
^1^PLANT= plant-based diet
^2^MEAT=animal-based diet
^a^Denotes a significant increase in metabolite concentration over time
^b^Denotes higher concentration in the PLANT group compared to the MEAT group
