# Supplementary material for: The fecal metabolomic signature of a plant-based (vegan) diet compared to an animal-based diet in healthy adult client-owned dogs
Source: J Anim Sci. 2025 Feb 27;103:skaf054. doi: 10.1093/jas/skaf054 (PMC12056932; doi:10.1093/jas/skaf054)
Supplement: skaf054_suppl_Supplementary_Figures_1-2_Tables_1-7 [file skaf054_suppl_supplementary_figures_1-2_tables_1-7.zip › Supplementary material Table 7_Nitrogen and other.docx]

**Table S7.** Nitrogenous bases and other metabolite concentrations quantified from the feces of 54 healthy adult dogs (n=25 neutered male, and n= 29 spayed female) participating in a randomized, double-blinded longitudinal study. Dogs were exclusively fed either a PLANT (n=30) or MEAT (n=24) diet for 3 months.

| **Metabolite** | **PLANT^1^ Baseline** | **PLANT^1^ Exit** | **MEAT^2^**  **Baseline** | **MEAT^2^**  **Exit** | **Association of PLANT^1^ over time**  **P-value** | **Association of MEAT^2^**  **over time**  **p-value** | **Association of PLANT^1^ vs MEAT^2^**  **Baseline**  **p-value** | **Association of PLANT^1^ vs MEAT^2^**  **Exit**  **p-value** |
| --- | --- | --- | --- | --- | --- | --- | --- | --- |
| **Nitrogenous bases metabolites** | | | | | | | | |
| Choline | 0.34  (0.00-1.08) | 0.39  (0.04-0.79) | 0.37  (0.18-0.64) | 0.35  (0.00-0.89) | 0.03 | 0.80 | 1.00 | 0.04^b^ |
| Acetoin | 0.00  (0.00-0.99) | 0.00  (0.00-0.58) | 0.00  (0.00-0.19) | 0.00  (0.00-7.37) | 0.79 | 0.63 | 0.92 | 0.99 |
| Formate | 0.69  (0.25-1.72) | 0.72  (0.35-16.82) | 0.73  (0.25-1.56) | 0.70  (0.36-1.51) | 0.09 | 1.00 | 1.00 | 0.57 |
| **Other metabolites** | | | | | | | | |
| Uracil | 6.47  (2.27-21.34) | 5.50  (1.03-11.45) | 7.43  (3.11-15.62) | 6.33  (0.86-12.34) | 0.03^a^ | 0.41 | 0.84 | 1.00 |
| Xanthine | 0.09  (0.03-1.49) | 0.11  (0.02-0.96) | 0.07  (0.03-0.61) | 0.08  (0.02-0.47) | 0.16 | 0.10 | 0.52 | <0.0001^b^ |
| Hypoxanthine | 2.07  (0.89-5.83) | 2.19  (0.47-3.42) | 2.06  (0.93-5.37) | 1.89  (0.17-6.15) | 0.63 | 0.30 | 0.73 | 0.44 |

Evaluation of interactions between diet and time were made between the two diet groups per timepoint and between two time-points within diet groups using mixed model gamma linear regression controlling for age, sex, and BW.
Data was presented as non-parametric metabolite concentrations between diet group at each timepoint are presented as median and interquartile range [minimum and maximum]. ^1^PLANT= plant-based diet
^2^MEAT=animal-based diet
^a^Denotes significant decrease in metabolite concentration over time
^b^Denotes higher concentration in the PLANT group compared to the MEAT group
