# Supplementary material for: The fecal metabolomic signature of a plant-based (vegan) diet compared to an animal-based diet in healthy adult client-owned dogs
Source: J Anim Sci. 2025 Feb 27;103:skaf054. doi: 10.1093/jas/skaf054 (PMC12056932; doi:10.1093/jas/skaf054)
Supplement: skaf054_suppl_Supplementary_Figures_1-2_Tables_1-7 [file skaf054_suppl_supplementary_figures_1-2_tables_1-7.zip › Supplementary Materials T4_FA.docx]

Table S4. Fatty acid metabolite concentrations quantified from the feces of 54 client-owned healthy adult dogs (n=25 neutered male, and n= 29 spayed female) participating in a randomized, double-blinded longitudinal study. Dogs were exclusively fed either a PLANT (n=30) or MEAT (n=24) diet for 3 months.

| **Metabolite** | **PLANT^1^ Baseline** | **PLANT^1^**  **Exit** | **MEAT^2^ Baseline** | **MEAT^2^**  **Exit** | **Association of PLANT^1^ over time**  **P-value** | **Association of MEAT^2^ over time**  **p-value** | **Association of PLANT^1^ vs MEAT^2^ Baseline**  **p-value** | **Association of PLANT^1^ vs MEAT^2^ Exit**  **p-value** |
| --- | --- | --- | --- | --- | --- | --- | --- | --- |
| Acetic acid | 294.57  (85.08-462.11) | 274.50  (130.03-480.05) | 254.87  (125.51-480.05) | 236.59  (113.68-409.59) | 0.15 | 0.17 | 0.29 | <0.001^c^ |
| Propionate | 148.54  (44.60-317.70) | 171.47  (10.69-399.90) | 133.97  (51.36-180.09) | 119.54  (39.45-285.14) | <0.0001^b^ | 0.25 | 0.33 | <0.0001^c^ |
| Butyrate | 47.81  (2.20-184.02) | 32.82  (2.04-156.72) | 48.85  (2.14-92.79) | 36.72  (8.03-196.99) | <0.001^a^ | <0.001^a^ | <0.001^c^ | <0.001^d^ |
| Valerate | 3.00  (0.67-9.52) | 3.75  (1.17-30.09) | 2.31  (1.26-30.09) | 2.37  (1.12-12.52) | 0.05^b^ | 0.93 | 1.00 | <0.001^c^ |
| 3-hydroxyisovaleric acid | 0.31  (0.08-0.72) | 0.35  (0.17-0.66) | 0.28  (0.13-0.55) | 0.23  (0.10-0.47) | 0.78 | 1.00 | 0.40 | 0.18 |
| Isobutyric acid | 5.32  (1.18-10.05) | 4.85  (1.13-19.41) | 4.45  (1.18-11.36) | 4.29  (2.65-9.48) | 1.00 | 0.89 | 0.97 | 0.74 |
| Isovaleric acid | 3.89  (0.96-11.79) | 3.09  (0.97-9.23) | 3.18  (1.11-9.23) | 3.15  (1.68-7.93) | <0.0001^a^ | 1.00 | 0.88 | 0.26 |
| Fumaric acid | 0.42  (0.07-1.08) | 0.30  (0.05-1.02) | 0.54  (0.25-1.56) | 0.43  (0.11-3.09) | 0.32 | 1.00 | 1.00 | 0.96 |
| Methylmalonic acid | 0.67  (0.03-2.17) | 0.35  (0.02-1.79) | 0.74  (0.03-2.52) | 0.40  (0.03-1.61) | 1.00 | 0.66 | 0.23 | 0.95 |
| Pyruvic acid | 0.28  (0.11-1.18) | 0.36  (0.10-1.21) | 0.34  (0.07-0.89) | 0.27  (0.05-9.97) | 0.94 | 0.74 | 1.00 | 0.93 |
| Malonate | 1.34  (0.41-3.52) | 1.28  (0.42-2.87) | 1.06  (0.48-1.64) | 0.94  (0.62-2.28) | 0.98 | 0.99 | 0.10 | 0.10 |

Evaluation of interactions between diet and time were made between the two diet groups per timepoint and between two time-points within diet groups using mixed model gamma linear regression control for age, sex, and BW.

As data was presented as non-parametric metabolite concentrations between diet group at each timepoint are presented as Median and interquartile range (minimum and maximum).

^1^PLANT= plant-based diet
^2^MEAT=animal-based diet
^a^Denotes significant decrease in metabolite concentration over time
^b^Denotes a significant increase in metabolite concentration over time
^c^Denotes higher concentration in the PLANT group compared to the MEAT group
^d^Denotes lower concentration in the PLANT group compared to the MEAT group
