# Supplementary material for: The fecal metabolomic signature of a plant-based (vegan) diet compared to an animal-based diet in healthy adult client-owned dogs
Source: J Anim Sci. 2025 Feb 27;103:skaf054. doi: 10.1093/jas/skaf054 (PMC12056932; doi:10.1093/jas/skaf054)
Supplement: skaf054_suppl_Supplementary_Figures_1-2_Tables_1-7 [file skaf054_suppl_supplementary_figures_1-2_tables_1-7.zip › Supplemnental Table 1_Demographics.docx]

Table S1. Demographics of 54 healthy adult client-owned dogs fed an experimental dry vegan diet (PLANT n=30) or commercial animal-based (MEAT n=24) extruded kibble diet in a 12-week randomized, double blinded longitudinal study, including breed, diet, age, body weight (BW), body condition score (BCS), and sex (spayed female n=29; neutered male n=25).

| **Dog ID** | **Breed** | **Diet** | **Age** | **BW^1^** | **BCS^2^** | **Sex** |
| --- | --- | --- | --- | --- | --- | --- |
| 1 | Labrador retriever | PLANT^4^ | 6 | 31.5 | 5 | Spayed female |
| 2 | akbash | PLANT^4^ | 7 | 48.3 | 5 | Neutered male |
| 3 | Labrador retriever | PLANT^4^ | 4 | 34.7 | 5 | Neutered male |
| 4 | Labrador retriever | PLANT^4^ | 3 | 28.6 | 5 | Neutered male |
| 5 | cattle dog x^3^ | PLANT^4^ | 9 | 43.5 | 6 | Neutered male |
| 6 | mix | PLANT^4^ | 8.5 | 21.1 | 5 | Spayed female |
| 7 | German shepherd x^3^ | PLANT^4^ | 7 | 27.3 | 5 | Spayed female |
| 8 | mastif x^3^ | PLANT^4^ | 8 | 35.8 | 5 | Neutered male |
| 9 | golden retriever | PLANT^4^ | 5 | 24.9 | 5 | Neutered male |
| 10 | Unknown mix | PLANT^4^ | 9.5 | 19.9 | 5 | Spayed female |
| 11 | coton de tulear x^3^ | PLANT^4^ | 4 | 6.3 | 6 | Spayed female |
| 12 | German shepherd x^3^ | PLANT^4^ | 7 | 20.9 | 5 | Spayed female |
| 13 | potcake | PLANT^4^ | 3 | 21.1 | 4 | Spayed female |
| 14 | labradoodle | PLANT^4^ | 3 | 24.9 | 6 | Spayed female |
| 15 | Australian shepherd | PLANT^4^ | 3 | 24.9 | 4 | Neutered male |
| 16 | Labrador retriever x^3^ | PLANT^4^ | 5 | 26.4 | 6 | Spayed female |
| 17 | cockapoo | PLANT^4^ | 3 | 7.1 | 6 | Neutered male |
| 18 | boxer x^3^ | PLANT^4^ | 8 | 26.8 | 6 | Spayed female |
| 19 | German shepherd x^3^ | PLANT^4^ | 3 | 20 | 5 | Spayed female |
| 20 | terrier x^3^ | PLANT^4^ | 7 | 5.5 | 5 | Neutered male |
| 21 | border collie | PLANT^4^ | 3 | 23.8 | 6 | Neutered male |
| 22 | golden retriever | PLANT^4^ | 3 | 26.9 | 5 | Spayed female |
| 23 | German shepherd | PLANT^4^ | 4 | 30.5 | 4 | Spayed female |
| 24 | potcake | PLANT^4^ | 3 | 21.1 | 4 | Neutered male |
| 25 | Australian shepherd | PLANT^4^ | 3 | 17.1 | 5 | Spayed female |
| 26 | German shepherd | PLANT^4^ | 3 | 27.7 | 5 | Spayed female |
| 27 | jack russel terrier | PLANT^4^ | 5 | 8.1 | 6 | Neutered male |
| 28 | hound mix | PLANT^4^ | 3 | 26.9 | 5 | Neutered male |
| 29 | mix | PLANT^4^ | 4 | 24.5 | 4 | Neutered male |
| 30 | German shepherd x^3^ | PLANT^4^ | 3 | 32.6 | 5 | Spayed female |
| 31 | pug x^3^ | MEAT^5^ | 4 | 9.4 | 6 | Spayed female |
| 32 | Labrador retriever | MEAT^5^ | 7 | 29.8 | 5 | Spayed female |
| 33 | Irish wolfhound | MEAT^5^ | 4 | 47.1 | 5 | Spayed female |
| 34 | bichon x^3^ | MEAT^5^ | 3.5 | 6.9 | 6 | Neutered male |
| 35 | golden retriever | MEAT^5^ | 5 | 34.8 | 5 | Neutered male |
| 36 | poodle | MEAT^5^ | 3 | 17.7 | 4 | Spayed female |
| 37 | Australian shepherd | MEAT^5^ | 9.5 | 20.2 | 6 | Neutered male |
| 38 | cattle dog x^3^ | MEAT^5^ | 8 | 28.7 | 6 | Neutered male |
| 39 | Labrador retriever | MEAT^5^ | 4.5 | 27.7 | 6 | Spayed female |
| 40 | Australian shepherd | MEAT^5^ | 6 | 23.3 | 6 | Spayed female |
| 41 | soft-coated Wheaten terrier | MEAT^5^ | 3 | 20.6 | 6 | Neutered male |
| 42 | klee kai | MEAT^5^ | 5 | 6.5 | 5 | Neutered male |
| 43 | Anatolian shepherd | MEAT^5^ | 3 | 38.3 | 5 | Neutered male |
| 44 | Yorkshire terrier | MEAT^5^ | 5 | 5.1 | 5 | Spayed female |
| 45 | Labrador retriever x^3^ | MEAT^5^ | 6 | 33.6 | 6 | Neutered male |
| 46 | Australian shepherd | MEAT^5^ | 8 | 19.4 | 5 | Spayed female |
| 47 | border collie | MEAT^5^ | 5 | 18.6 | 5 | Spayed female |
| 48 | schnauzer | MEAT^5^ | 6 | 7.3 | 5 | Spayed female |
| 49 | Labrador retriever x^3^ | MEAT^5^ | 3 | 32.9 | 6 | Neutered male |
| 50 | golden retriever | MEAT^5^ | 4 | 28.9 | 5 | Spayed female |
| 51 | klee kai | MEAT^5^ | 3 | 9.1 | 6 | Neutered male |
| 52 | springer spaniel | MEAT^5^ | 3 | 12.5 | 5 | Spayed female |
| 53 | German shepherd | MEAT^5^ | 5 | 30.4 | 5 | Spayed female |
| 54 | boxer | MEAT^5^ | 3 | 31.9 | 5 | Neutered male |

^1^BW, body weight,
^2^BCS, body condition score
^3^x, crossbreed
^4^PLANT, plant-based diet,
^4^MEAT, animal-based diet
