# Supplementary figures and images for: The fecal metabolomic signature of a plant-based (vegan) diet compared to an animal-based diet in healthy adult client-owned dogs
Source: J Anim Sci. 2025 Feb 27;103:skaf054. doi: 10.1093/jas/skaf054 (PMC12056932; doi:10.1093/jas/skaf054)

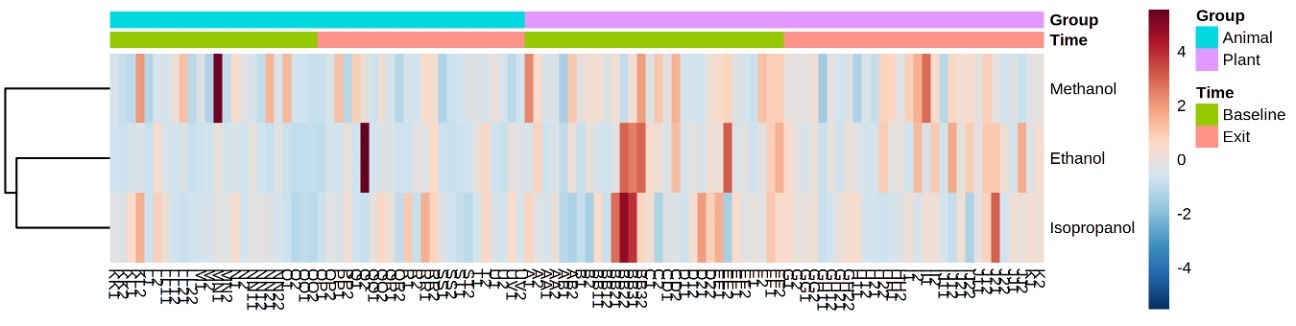

Supplement: skaf054_suppl_Supplementary_Figures_1-2_Tables_1-7 [file skaf054_suppl_supplementary_figures_1-2_tables_1-7.zip › Supplemental_Fig_1 (1).jpg]

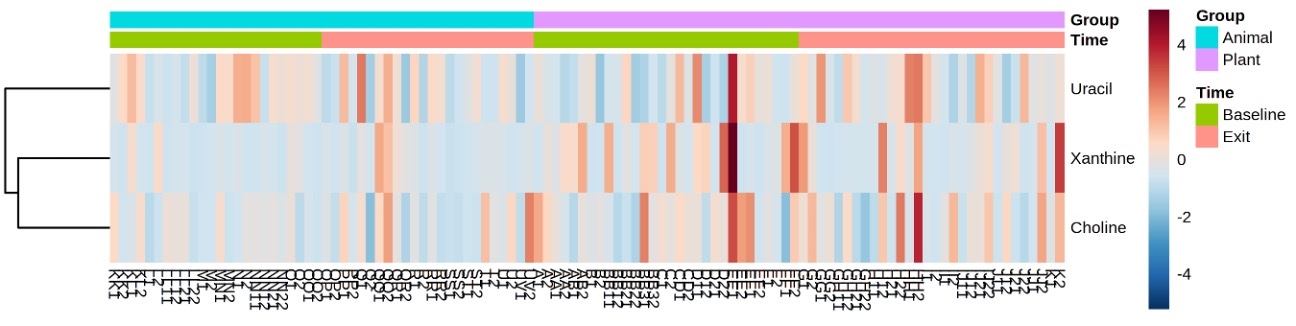

Supplement: skaf054_suppl_Supplementary_Figures_1-2_Tables_1-7 [file skaf054_suppl_supplementary_figures_1-2_tables_1-7.zip › Supplemental_Fig_2.jpg]
